# Antiretroviral treatment outcome in HIV-1-infected patients routinely followed up in capital cities and remote areas of Senegal, Mali and Guinea-Conakry

**DOI:** 10.7448/IAS.17.1.19315

**Published:** 2014-12-18

**Authors:** Abou Abdallah Malick Diouara, Halimatou Diop Ndiaye, Ibrehima Guindo, Nestor Bangoura, Mohamed Cissé, Tchiakpe Edmond, Flabou Bougoudogo, Souleymame Mboup, Martine Peeters, Ahidjo Ayouba, Ndèye Coumba Touré Kane

**Affiliations:** 1 Laboratoire de Bactériologie Virologie CHU Aristide Le Dantec, Université Cheikh Anta Diop de Dakar, Dakar, Sénégal; 2 Service de Bactériologie-Virologie Institut, National de Recherche en Santé Publique (INRSP) de Bamako, Bamako, Mali; 3 Service de Dermatologie CHU Donka, CTA, Conakry, République de Guinée; 4 UMI 233 TransVIHMI, IRD and Université de Montpellier 1, Montpellier, France

**Keywords:** HIV-1 drug resistance, viral load, HIV-1 genetic diversity, dried blood spots, remote areas, West Africa

## Abstract

**Introduction:**

Access to antiretroviral treatment (ART) becomes more and more effective in resource-limited settings (RLS). However, this global effort would be even more profitable if the access to laboratory services especially in decentralized settings was strengthened. We report the virological outcome and HIV-1 drug resistance in three West African countries using dried blood spots (DBS) samples.

**Methods:**

We included HIV-1-infected adults on ART ≥6 months and followed up in capital cities and decentralized sites in Senegal, Mali and Guinea-Conakry. Patients were consecutively enrolled and DBS were collected in field conditions and kept at ambient temperature before transfer to the reference laboratory. Viral load (VL) was quantified using the NucliSENS EasyQ HIV-1 v1.2. Genotyping of HIV-1 *pol* gene was performed using in-house protocol.

**Results:**

Of the 407 participants, 119, 152 and 136 were from Senegal, Mali and Guinea-Conakry, respectively. The median treatment duration was 36 months [IQR: 6–136]. Virological failure (VF) (VL≥3log_10_ copies/mL) was observed in 26% (95% confidence interval (CI), 18–35; n=31), 11% (95% CI, 6–17; n=16) and 24% (95% CI, 17–32; n=33) of patients in Senegal, Mali and Guinea-Conakry, respectively (*p*=0.001). Of samples presenting VL≥3log_10_ copies/mL (n=80), 70 were successfully genotyped. At least one drug resistance mutation (DRM) was detected in the following proportions: 70% (95% CI, 50–86; n=19), 93% (95% CI, 68–100; n=14) and 68% (95% CI, 48–84; n=19) in Senegal, Mali and Guinea-Conakry, respectively (*p*=0.22). Twenty-six per cent (26%; 95% CI, 16–38; n=18) of patients in VF harboured wild-type viruses, which is likely indicative of weak adherence. Phylogenetic analysis showed the predominance of CRF02_AG subtype (73%; 95% CI, 61–83; n=51).

**Conclusions:**

We describe the ART outcome in capital and rural settings of Senegal, Mali and Guinea-Conakry. Our results in all of the three countries highlight the need to reinforce the ART adherence in order to minimize the occurrence of drug resistance. In addition, these findings provide additional evidence that the use of DBS as a sampling support could assist virological monitoring of patients on ART in remote areas.

## Introduction

In the latest UNAIDS report, the number of people on antiretroviral therapy (ART) has dramatically increased in the past few years [1]. However, ART expansion was not sufficiently accompanied by access to laboratory services and diagnostics, especially in remote areas of developing countries. It is also established that now decentralizing HIV care at the community level is an essential link for the retention of patients in the healthcare system [2,3], which, in addition, promotes good treatment adherence. Consequently, it determines successful long-term viral load (VL) suppression [4]. HIV VL and resistance testing are essentials for monitoring the response to treatment, diagnosing and confirming treatment failure (TF) and surveillance of drug resistance. However, in remote areas of developing countries lacking appropriate equipment for plasma processing and transportation, virological monitoring is still a challenge. Several studies have demonstrated that the filter paper is a suitable tool for blood sample collection, transportation and storage [5–11] and may facilitate HIV virological monitoring. However, few studies have reported the use of dried blood spots (DBS) in routine field conditions to account with variable environmental conditions [11–14]. The aim of this study was to document the virological outcome and HIV-1 drug resistance in adult patients followed in the capital cities and decentralized settings in three West African countries, namely Senegal, Mali and Guinea-Conakry, using DBS samples.

## Materials and methods

### Study design and settings

We conducted a multi-site study on patients on ART in capital cities and decentralized medical centres of three West African countries (Senegal, Mali and Guinea-Conakry). We included patients who were at least 18 years old, under ART treatment for at least six months and consenting to participate in the study. Women who received the protocol for the prevention of mother-to-child transmission (PMTCT) as well as patients positive for HIV-2 or co-infected (HIV-1 and HIV-2) were not included. Patients were consecutively enrolled between February 2010 and December 2011 in 17 sites distributed as follows: Senegal (n=7), Mali (n=6) and Guinea-Conakry (n=4). In Mali and Guinea-Conakry, recruitment sites were chosen depending on the active file of patients. In Senegal, all samples received for routine virological monitoring, from patients who met the inclusion criteria were considered.

### Ethical considerations

National Ethics Committees of participating countries approved this study. Patients were recruited on a voluntary basis. To keep confidential data of the participants, a unique identifier was assigned to each sample and used throughout the study, ascertaining anonymity. Only the attending physician could establish the correspondence between this identifier and the patient.

### DBS preparation and shipping

For each patient, two DBS cards (Whatman 903 filter paper, Dassel, Germany) were spotted: 50 µL/spot, five spots/card from whole blood EDTA tube. Cards were left to dry overnight at ambient temperature (range 22–37°C) before being packed in an individual sealed bag with desiccants and a humidity indicator card. These individual bags were in turn packed in zip-lock plastic bag and sent at ambient temperature to the reference laboratory in Dakar, Senegal, for testing. On site, collected DBS specimens were checked frequently for humidity and sent within one month after sampling. At the reference laboratory, upon reception, the conformity (in respect of delivery times, presence of humidity indicator card, desiccant packets and integrity of blood spots [no moisture]) was checked and DBS were stored at −80°C until testing. The samples were discarded if they did not meet the criteria mentioned above.

### Laboratory procedures

Total HIV-1 nucleic acids were extracted from two spots with NucliSENS miniMAG (bioMérieux, Craponne, France) with magnetic silica as previously described [5]. Briefly, two spots from each sample were punched and placed in a tube containing 2 mL of lysis buffer. After 30 min of gentle rocking at room temperature, the supernatant was clarified by centrifugation at 2500 rpm during two minutes and then transferred to a clean 15 mL conical tube. Extracted nucleic acids were eluted in 25 µL of elution buffer and stored at 4°C for immediate use (VL quantitation or PCR amplification) or at −80°C for longer storage.

HIV-1 VL was quantified using the NucliSENS EasyQ HIV-1 v1.2 (bioMérieux, Marcy l'Etoile, France) according to manufacturers’ instructions. The VL cut-off was 800 copies/mL with this assay for DBS [15]. In the present study, we set the VF to 3log_10_ (1000) copies/mL as recently recommended by WHO [4].

HIV-1 drug resistance test was performed according to the ANRS AC11 protocol (http://www.hivfrenchresistance.org/) by amplifying separately the entire *Protease* (PR) gene and first 240 codons of *Reverse Transcriptase* (RT) using, respectively, 5′Prot1/3′Prot1 and MJ3/MJ4 as outer primers and 5′Prot2/3′Prot2 and A35/NE35 as inner primers. Second round PCR products were purified with QIAquick Gel Extraction Kit^®^ (Qiagen, Courtaboeuf, France) according to the manufacturers’ instructions. Purified DNA was sequenced directly on ABI 3100 Avant Genetic Analyzer using Big Dye Terminator Technology^®^v3.1 (Applied Biosystems, Carlsbad, CA) and their respective inner primers. Sequences obtained were assembled and edited manually using SeqMan™ II 5.08 from DNAstar^®^software (Lasergene, Konstanz, Germany). Drug resistance analysis and interpretation were performed using the Stanford University HIV database version 6.0.8 (http://hivdb.stanford.edu/). HIV-1 subtypes were determined by phylogeny. Nucleotide sequences were aligned with a set of reference sequences of HIV-1 group M subtype and circulating recombinant forms (CRFs) downloaded from Los Alamos HIV database (http://www.hiv.lanl.gov/content/index). Each subtype was represented by at least three reference sequences. Sequences were aligned with MUSCLE (and gap positions removed by using Gblocks program on SEAVIEW v4.4.1). Maximum Likelihood phylogeny was inferred online using the PhyML software (http://www.atgc-montpellier.fr/phyml) with branch supports determined by the approximate likelihood ratio test method (aLRT) SH-like option, and the substitution model was GTR+I+G. The recombinant strains analysis (similarity and bootscanning) were performed on Simplot software v3.5.1 [16].

The new *PR* and *RT* generated sequences were deposited in EMBL with the following accession Numbers: HG380024 to HG380051, HG380054 to HG380063, HG380065 to HG380069, HG424394 to HG424413, HG424415, HG424417, HG424418 and HG424420 to HG424423.

### Statistical analysis

Data were analyzed using Epi Info™ Version 3.5.3. Ninety-five per cent confidence intervals (CIs) were used for all estimates. The chi-square test was used with Yate's correction to search the link between the variables. Comparisons between median values of VL of amplified samples or not were performed using Mann–Whitney test. P values less than 0.05 were considered statistically significant.

## Results

Of the 407 patients enrolled at 17 collection sites in three West African countries, 93% (95% CI, 90–95; n=379) were on first-line ART (2NRTI+1NNRTI) with zidovudine (AZT), lamivudine (3TC), stavudine (D4T), nevirapine (NVP) and efavirenz (EFV) the most commonly used (85%; 95% CI, 81–88; n=346). Patients on second-line ART (2 NRTI+1 boosted-PI) (7%; 95% CI, 5–10; n=28) were distributed as follows: Senegal (n=5), Mali (n=16) and Guinea-Conakry (n=7). The ART median follow-up duration was 36 months [IQR: 6–136], with a higher proportion of females (69%; 95% CI, 64–73; n=279), (*p*=0.03) and the median age of the study population was 40 years [IQR: 18–66]. Table 1 summarizes the demographic and biological data of patients per country.

**Table 1:** Demographic and biological data of patients per country

Countries	Senegal	Mali	Guinea-Conakry	Total
Sample collection sites	7	6	4	17
Number of patients enrolled	119	152	136	407
Female (%)	94 (79%)	102 (67%)	83 (61%)	279 (69%)
Median age (years)	42 [IQR: 18–65]	41 [IQR: 18–66]	38 [IQR: 18–61]	40 [IQR: 18–66]
First-line therapy (2 NRTI+1 NNRTI)	114 (96%)	136 (89%)	129 (95%)	379 (93%)
AZT+3TC+NVP/EFV	109	66	80	255
D4T+3 TC+NVP/EFV	1	43	47	91
Other first-line combinations	4	27	2	33
Second-line therapy (2 NRTI+1 PI)	5	16	7	28
Median time on ART	32 [IQR: 6–112]	39 [IQR: 6–136]	35 [IQR: 6–108]	36 [IQR: 6–136]
VL>technical cut-off (800 copies/mL)	31	17	33	81
Median of viral load	3.63 [IQR: 3–5.48]	3.94 [IQR: 2.97–6.18]	3.64 [IQR: 3.07–6.75]	3.68 [IQR: 2.97–6.75]
Virological failure (VL>3log_10_ copies/mL)	31 (26%)	16(11%)	33 (24%)	80 (20%)
Genotyped	27 (87%)	15 (94%)	28 (85%)	70 (88%)
Any DRM	19	14	19	52
DRM in patients with virological failure	70% (n=19/27)	93% (n=14/15)	68% (n=19/28)	74% (n=52/70)
Global DRM	16% (n=19/119)	9% (n=14/152)	14% (n=19/136)	13% (n=52/407)

AZT: zidovudine; D4T: stavudine; NVP: nevirapine; 3TC: lamivudine; EFV: Efavirenz; ART: antiretroviral therapy; NNRTI: non-nucleoside reverse transcriptase inhibitor; NRTI: nucleoside reverse transcriptase inhibitor; PI: protease inhibitor; VL: viral load; DRM: drug resistance mutation.

### Specificities of participating countries

#### Senegal

In Senegal, 119 patients were included. Recruitment took place at six regional medical centres [Louga (n=27), Thiès (n=18), Diourbel (n=13), Fatick (n=8), St-Louis (n=2), Ziguinchor (n=2)] and one in the suburb of Dakar [Roi Baudouin (n=49)]. The median age was 42 years [IQR: 18–65] and 79% (95% CI, 71–85; n=94) of patients were female. Among the 119 patients, 96% (95% CI, 90–99; n=114) were on first-line ART mainly with AZT+3TC+NVP/EFV combination 96% (95% CI, 90–99; n=109) (Table 1). The median duration of ART was 32 months [IQR: 6–112]. The substitution of AZT by TDF had occurred in two cases.

A total of 26% (95% CI, 18–35; n=31) of the patients had detectable VL (≥800 copies/mL). All of them were in VF (VL≥3log_10_ copies/mL) and the median VL was 3.63log_10_ copies/mL [IQR: 3–5.48]. According to the treatment duration, patients in VF were distributed as follows: 19.2% (95% CI, 7–39; n=5), 39.1% (95% CI, 22–59; n=9) and 24.2% (95% CI, 15–36; n=70), respectively, at 6–12, 13–24 and >24 months (*p*=0.24). Furthermore, data from Table 2 show that 45% (95% CI, 27–64; n=14) of patients in VF had VL higher than 3.7log_10_ copies/mL and were distributed as follows, according to treatment duration: 60% (95% CI, 15–95; n=3), 44% (95% CI, 14–79; n=4) and 41% (95% CI, 18–67; n=7), respectively, at 6–12, 13–24 and >24 months. Among the patients in the group >24 months (n=17), three were on second-line ART. Based on medical centres (n=7), no difference was found in the occurrence of VF between patients followed in Dakar, the capital city (24%; 95% CI, 13–39; n=12), and those of the regional centres (27%; 95% CI, 17–39; n=19), (*p*=0.91).

**Table 2:** Viral load (VL) and drug resistance mutation (DRM) distribution in patients in virological failure (VF) according to treatment duration

	Senegal			
	
Treatment duration	M6–M12	M13–M24	>M24	Total
Sample size	26	23	70	119
VF (VL>3log_10_ copies/mL)	5	9	17	31
VF (VL>3.7log_10_ copies/mL)	3	4	7	14
Genotyped	4	7	16	27
Any DRM	1	4	14	19
No DRM	3	3	2	8
**Mali**				
Sample size	22	27	103	152
VF (VL>3log_10_ copies/mL)	1	3	12	16
VF (VL>3.7log_10_ copies/mL)	0	3	7	10
Genotyped	1	3	11	15
Any DRM	1	3	10	14
No DRM	0	0	1	1
**Guinea-Conakry**				
Sample size	13	31	92	136
VF (VL>3log_10_ copies/mL)	4	7	22	33
VF (VL>3.7log_10_ copies/mL)	2	2	11	15
Genotyped	4	5	19	28
Any DRM	3	2	14	19
No DRM	1	3	5	9

**Figure 1:**
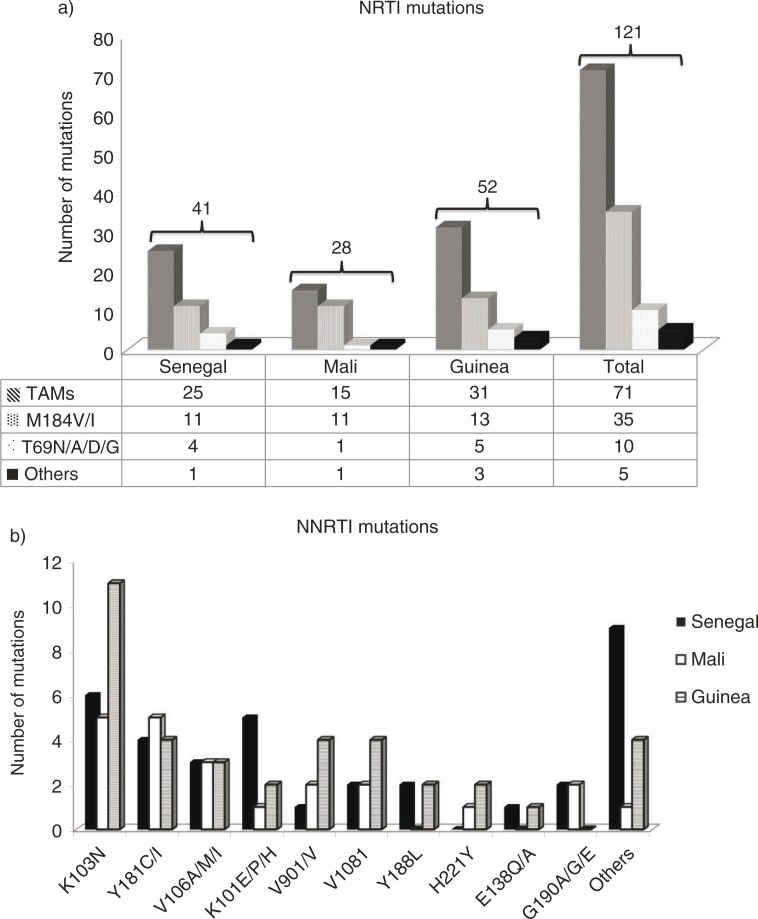
a) NRTI's resistance mutation prevalence. b) NNRTI's resistance mutations prevalence.

Of the samples with VL≥3log_10_ copies/mL, a genotypic test was successfully performed in 87% (95% CI, 70–96; n=27). The median VL of genotyped samples was 3.6log_10_ copies/mL [IQR: 3–5.4] and those not amplified (n=4) presented VL of: 3, 3.1, 3 and 3.4log_10_ copies/mL. Resistance analysis among 27 amplified samples showed that at least one drug resistance mutation (DRM) was found in 19 cases. Global resistance rate was 16% (95% CI, 10–24; n=19). Figure 1 shows the detailed numbers of NRTI- and NNRTI-associated DRMs. Forty-one NRTI-associated DRMs were observed in 52% (95% CI, 32–71; n=14) of patients and the most common mutation encountered was the M184V/I (27%; 95% CI, 14–43; n=11). Thymidine-associated mutations (TAMs) were found in 61% (95% CI, 45–76; n=25). Four patients had T69 insertion complex conferring resistance to all NRTIs currently approved by the USA FDA. With the exception of one patient with subtype B virus and treated by d4T+3TC+EFV, all patients with T69 mutation harboured CFR02_AG viruses and were on AZT+3TC+NVP/EFV. The NNRTI-associated DRMs were found in 67% (95% CI, 46–83; n=18). The most prevalent NNRTI-associated DRM was K103N detected in seven cases followed by K101E/P/H mutation carried by five patients. Of interest, 68% (95% CI, 43–87; n=13) of patients harboured NRTIs and NNRTI-associated mutations implying a dual class resistance. One patient on AZT+3TC+LPV/r and previously treated by FTC+TDF+LPV/r association had both PI-associated DRMs (G48M, I54V, L76V and V82A) and NRTI-associated DRMs.

Among the 27 genotyped samples, 30% (95% CI, 14–50; n=8) presented no DRM (Table 2) with three, three and two of them being on ART for 6–12, 13–24 and >24 months, respectively (Table 2).

**Figure 2:**
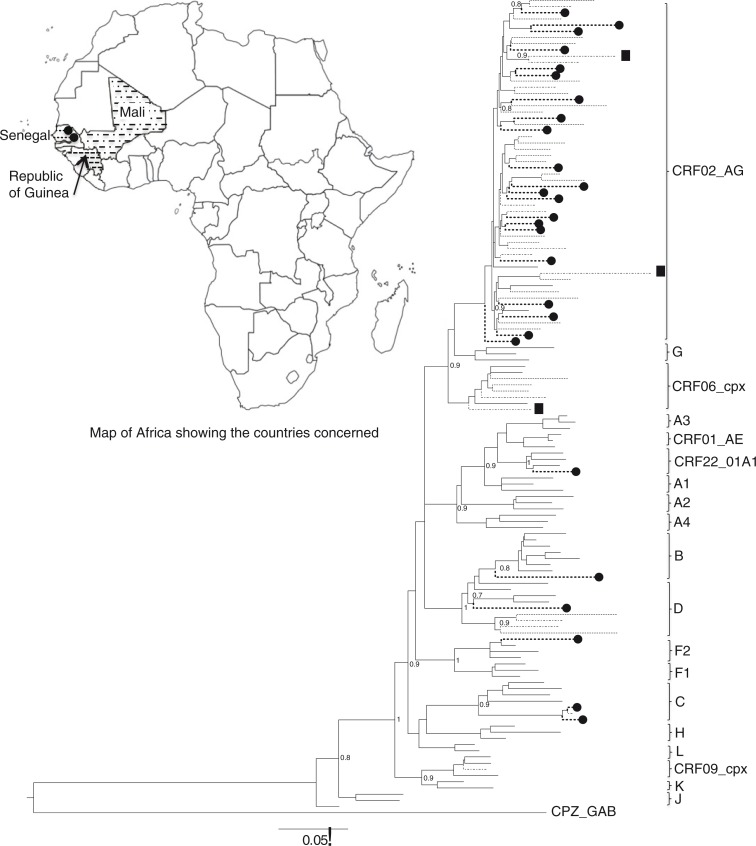
Maximum likelihood tree showing the relationships between pol sequences isolates from Senegal (-------●), Mali (-·-·-) and Guinea-Conakry (------). The corresponding legends are used to localise each country on the map. # Designate URFs. The reference strains are in continuous lines. The tree was constructed under the GTR+I+G model of evolution using PhyML on online program http://www.atgc-montpellier.fr/phyml.

Phylogenetic analysis (Figure 2) shows the predominance of CRF02_AG (78%; 95% CI, 58–91; n=21). Subtype C was found in two cases and other subtypes were observed in single occurrence: B, D, F2 and CRF22_01A1.

#### Mali

In Mali, 152 patients were enrolled at six sites [USAC Commune (n=30), Hôpital Sikasso (n=30), USAC Kati (n=29), CSRef Bougouni (n=28), ARCAD Mopti (n=20) and Clinique de santé sexuelle (n=15)] distributed in four regional medical centres [Bamako (n=47), Koulikoro (n=29), Mopti (n=30) and Sikasso (n=58)]. The median age was 41 years [IQR: 18–66] and 67% (95% CI, 59–75; n=102) were female (Table 1). Most of the patients (89%; 95% CI, 83–94; n=136) were on first-line ART (2NRTI+1NNRTI) and 11% (95% CI, 6–17; n=16) received one boosted-PI+2 NRTI. The ART median time was 39 months [IQR: 6–136].

For patients on first-line ART regimen, AZT+3TC+NVP/EFV (49%; 95% CI, 40–57; n=66) and d4T+3TC+NVP/EFV (32%; 95% CI, 24–40; n=43) combinations were most frequently observed (Table 1). For some patients, because of toxicity and/or drug stock-out, substitutions cases had occurred, for example, d4T by AZT (26%; 95% CI, 19–35; n=36) and AZT or d4T by TDF (12%; 95% CI, 7–19; n=17).

Seventeen patients (11%; 95% CI, 7–17; n=17) had detectable VL (≥800 copies/mL). The median of detectable VL was 3.9log_10_ copies/mL [IQR: 2.9–6.1]. The VF (VL≥3log_10_ copies/mL) was observed in 11% (95% CI, 6–17; n=16). According to the treatment duration, 5% (95% CI, 0–23; n=1), 11% (95% CI, 2–29; n=3) and 12% (95% CI, 6–19; n=12) of patients in VF were, respectively, at 6–12, 13–24 and >24 months (*p*=0.61) (Table 2). Among them, 62% (95% CI, 35–85; n=10) distributed as follows: zero, three and seven, respectively, at 6–12, 13–24 and >24 months of treatment, had a viremia higher than 3.7log_10_ copies/mL (Table 2). In a group of >24 months, three patients in VF were in second-line regimen. No difference was found in the occurrence of VF between patients followed in Bamako, the capital city (7%; 95% CI, 1–18, n=3), and those of the regional centres (12%; 95% CI, 7–20; n=13), (*p*=0.47).

Of the samples with VL≥3log_10_ copies/mL, resistance testing was successfully performed in 94% (95% CI, 70–100; n=15). The only one unamplified sample had a VL equal to 3.9log_10_ copies/mL. All amplified samples (n=15), except one, harboured at least one DRM representing global resistance rate of 9% (95% CI, 5–15; n=14) (Table 2). Resistance to both NRTIs and NNRTIs was observed in 86% (95% CI, 57–98; n=12) and one of them had in addition PI-resistance mutation (M46I). The multi-class resistance was found in this patient still naive to PI-containing regimen. Another patient had only NRTI-associated DRMs (V90I), which was on AZT+3TC+LPV/r and previously on TDF+FTC+EFV. PI-associated DRMs (M46I, I47V, L76V and I84V) and NNRTI-associated DRMs were found in one patient on ABC/3TC/LPV/r. Figure 1 shows details of DRM observed. Of the 28 NRTI-associated DRMs counted in 93% (95% CI, 68–100; n=14) amplified samples, M184V/I was the most represented (39%; 95% CI, 22–59; n=11). TAMs were also found in high proportion (54%; 95% CI, 34–72; n=15). For NNRTI-associated DRMs, K103N and Y181C were the most observed with 23% (95% CI, 8–45; n=5) each. One of the amplified samples presented no DRM (Table 2).

Phylogenetic analysis of 15 viral sequences showed a relative predominance of CRF02_AG 47% (95% CI, 21–73; n=7). Other subtypes were also found: D (n=2), CRF09_cpx (n=1), CRF06_cpx (n=1), C (n=1) and three URFs (CRF02_AG/CRF09_cpx/CRF02_AG, CRF02_AG/CRF06_cpx/CRF02_AG, and CRF02_AG/U/A3) (Figure 2).

#### Guinea-Conakry

In Guinea-Conakry, 136 patients were enrolled in this survey. Recruitment took place in Conakry, the capital city (CHU Donka, n=44) and three regional medical centres [Boké (n=33), Mamou (n=35), Labé (n=24)]. The median age of patients was 38 years [IQR: 18–61] and 61% (95% CI, 52–69; n=83) were female. Ninety-five per cent of patients (95%; 95% CI, 90–98; n=129) were on first-line ART (2NRTI+1NNRTI) and 5% (95% CI, 2–10; n=7) on second-line ART (2NRTI+1boosted-PI). The ART median time was 35 months [IQR: 6–108]. For patients undergoing first-line ART regimen, AZT+3TC+NVP/EFV (62%; 95% CI, 53–70; n=80) and d4T+3TC+NVP/EFV (36%; 95% CI, 28–45; n=47) combinations were most frequently observed (Table 1). The treatment history shows that in some patients, because of toxicity and/or drug stock-out, substitutions cases had occurred, for example, d4T by AZT (46%; 95% CI, 37–55; n=59) and AZT or d4T by TDF (3%; 95% CI, 1–8; n=4). Virological failure (VF) (VL≥3log_10_ copies/mL) was observed in 24% (95% CI, 17–32; n=33) and median VL was 3.6 [IQR: 3–6.7] log_10_ copies/mL. The VL distribution showed that 48% (95% CI, 31–66; n=16) of patients in VF had VL greater than 3.7log_10_ copies/mL. Stratified by treatment duration, 31% (95% CI, 9–61; n=4), 23% (95% CI, 10–41; n=7) and 24% (95% CI, 16–34; n=22) of patients in VF were, respectively, at 6–12, 13–24 and >24 months (*p*=0.22) (Table 2). In the groups of patients at 13–24 and >24 months of treatment duration, one and four were, respectively, on second-line ART. Based on medical centres (n=4), no difference was found in the occurrence of VF between patients followed in Conakry, the capital city (32%; 95% CI, 19–48; n=14), and those of the regional centres (21%; 95% CI, 13–30; n=19), (*p*=0.15).

Of the samples with VL≥3log_10_ copies/mL, genotypic tests were successfully performed in 85% (95% CI, 68–95; n=28). The median VL of genotyped samples (n=28) was 4 [IQR: 3–6.7] log_10_ copies/mL and those not amplified (n=5) was 3.1 [IQR: 3–3.6] log_10_ copies/mL (*p*=0.02). Nineteen of the 28 patients with available genotypic results harboured at least one DRM giving a global resistance rate of 14% (95% CI, 9–21; n=19). Resistance to both NRTIs and NNRTIs was observed in 79% (95% CI, 54–94; n=15), and four patients had only NNRTI-associated DRMs. Otherwise, no PI-associated DRM has been observed among the patients on first-line or among those on second-line ART. A total of 52 NRTI-associated DRMs were detected and the M184V/I mutation was found in 25% (95% CI, 14–39; n=13). TAMs were found in 60% (95% CI, 45–73; n=31). Five patients had T69 insertion complex conferring resistance to all NRTIs currently approved by the US FDA. All of them harboured CRF02_AG virus and were at >24 months of treatment. Their treatment regimens were: AZT+3TC+NVP/EFV (n=3), d4T+3TC+NVP (n=1) and AZT/3TC/LPVr (n=1). The most common NNRTI mutation was K103N (30%; 95% CI, 16–47; n=11) followed by Y181C/I, V90I/V and V108I. Each of these three mutations mentioned was found four times (Figure 1). No DRM was found in 32% (95% CI, 16–52; n=9) of amplified samples distributed as follows: 1, 3 and 5 respectively at 6–12, 13–24 and >24 months of treatment duration (Table 2).

The phylogenetic analysis shows that 82% (95% CI, 63–94; n=23) of patients were infected by CRF02_AG subtype. Subtypes D and CRF06_cpx have been found in the respective proportions of 7% (95% CI, 1–24; n=2) and 11% (95% CI, 2–28; n=3) (Figure 2 ).

## Discussion

The aim of this work was to document the virological outcome and HIV-1 drug resistance in adult patients followed in capital cities and decentralized settings in Senegal, Mali and Guinea-Conakry using DBS samples. Most of the patients in this study were on first-line ART regimen (93%; 95% CI, 90–95; n=379), with a wide predominance of AZT+3TC+NVP/EFV combination followed by d4T+3TC+NVP/EFV according to WHO 2006 recommendations for resource-limited settings (RLS) [17]. Similar observations were also reported in previous studies conducted in Senegal [18], Mali [19–21] and other sub-Saharan African countries [12,22,23]. Drug substitutions consisted mainly of d4T phasing out, which was replaced by AZT (36%; 95% CI, 30–42; n=95). Furthermore, substitutions occurred between AZT and TDF (9%; 95% CI, 6–13; n=24) according to WHO guidelines in 2009 [24].

Despite the difference in VL thresholds (800 copies/mL for “DBS” vs. 50 copies/mL for “Plasma”), the rate of viral suppression in our study (80%; 95% CI, 76–84; n=327) is comparable to those reported in multicentre studies in Burkina Faso and Mali (77.2%, n=467/606) [20], (74.9%, n=598/798) [25], (*p*=0.13). However, our findings show differences between the VF (VL≥3log_10_ copies/mL) rate noted in Senegal (26%; 95% CI, 18–35; n=31), Mali (11%; 95% CI, 6–17; n=16) and Guinea-Conakry (24%; 95% CI, 17–32; n=33), (*p*=0.01). This difference could be due partly to the size of the study populations between the three countries. In Senegal, all samples received routine virological monitoring and those who met the inclusion criteria were considered. Therefore, the number of patients failing treatment is not representative of the actual situation in the sub-population of persons on ART. Recently, a study outlining virological outcome in patients receiving HAART, and monitored with the World Health Organization (WHO) Public Health Approach, showed a VF of 10.7% among patients at 24 months’ treatment duration and recruited consecutively [26], which is significantly lower than 26% observed in the present study. Furthermore, without comparing health systems in order to correlate it to the virological outcome and resistance viruses, the differences observed could find explanation partly in the level of health care services between settings as reported by Pere *et al*. [23].

In Senegal, the study showed a similar VF rate (26%; 95% CI, 18–35; n=31) as previously reported by Diouara *et al*. (23.8%, n=55/231), (*p*=0.74) [13]. The differences seem linked to the size of the study populations, which is more important in this study. Another possible reason is the improvement of health care services and the expansion of virological monitoring (e.g. VL test) to decentralized level [27].

For Guinea-Conakry, to our knowledge, this is the first data of VL and antiretroviral drug resistance in HIV-1-treated patients. So, our results cannot be compared to the national level. However, VF is comparable to what was previously obtained in the neighbouring countries, namely Senegal and Mali [13,19]. Moreover, our findings show that there were no significant differences in the occurrence of VF between patients followed in capital cities and those in regional centres.

To the best of our knowledge, this is the first work in West Africa using DBS samples in VL and drug resistance testing. Overall, we had a good rate of PCR amplification (88%; 95% CI, 78–94; n=70), which is comparable to that obtained previously in Senegal [13] and those reported in other studies [6,11,14,28]. However, in Kenya, Arnedo *et al*. observed a lower PCR amplification rate (32.7%, n=18/55) [12]. Also, low PCR amplification rate (46%) was reported in a study comparing drug resistance profiles obtained from paired plasma and DBS samples from HIV-1-treated children living in Bangui, Central African Republic [29]. In addition, several studies showed a weak successful PCR amplification rate if VL<3.7log_10_ copies/mL [30]. This is in line with our results as it shows significant difference between medians VL of genotyped samples (n=70) and those not amplified (n=10), (*p*=0.03). It has also been reported in several studies that many factors, mainly the conditions and storage time, transportation, temperature, humidity level and genotyping methods could influence the efficiency of DBS samples PCR amplification [7,–9,11–14,31,32]. In addition, based on literature data and on our experience in usage of DBS samples collected in real-life conditions for drug resistance tests, in-house methods by amplifying and sequencing the entire *PR* and *RT* (at least codon 41–236) as described above seem to be most suitable in addition to the prerequisites for the conservation of sample integrity throughout the pre-analytical process [8,32]. In the present study, DBS transportation at an ambient temperature for a duration of 15–30 days, even when dealing with international shipments, do not seem to have any impact on the performance of amplification success. Similar observations were made by Parkin *et al*. in 2012 and recently by Parry *et al*. (2014). Their reports have also mentioned that the international shipment at an ambient temperature did not affect amplification success rates [11,33].

Of a total of 407 patients on ART according to WHO 2006 revised recommendations for RLS, 13% (95% CI, 10–16; n=52) had at least one DRM. Depending on the country, no significant difference was observed between the DRM rate: 70% (95% CI, 50–86; n=19) in Senegal, 93% (95% CI, 68–100; n=14) in Mali and 68% (95% CI, 48–84; n=19) in Guinea-Conakry, (*p*=0.15). The DRM profiles in these three countries were similar and show that TAMs (*p*=0.81), M184V/I (*p*=0.37) and K103N (*p*=0.45) mutations were by far the most frequently encountered. Their strong representation seems to be associated with the wide use of AZT/D4T+3TC+EFV/NVP in first-line protocol. Our results are in line with previous studies [22,23] reporting the accumulation of TAMs with treatment duration and frequency of the M184V and K103N mutations causing resistance to 3TC/FTC and NVP/EFV, respectively.

Overall, 26% (95% CI, 16–38; n=18) of genotyped samples were distributed as follows: Senegal (n=8), Mali (n=1) and Guinea-Conakry (n=9) had wild-type viruses (Table 2). This event could probably be related to poor adherence or a VF due to minority variants that could not be detected by bulk sequencing, as performed in the present study. However, similar observations were reported in a recent prospective cohort study conducted in Abidjan, Côte d'Ivoire, where 25% of patients in VF at month 24 of treatment harboured wild-type viruses [22]. Pere *et al*. in a study conducted in Bangui, Central African Republic, also reported that 24% of patients in VF showed wild-type viruses [23]. It is known that high VL on treatment is associated with a risk of developing drug resistance. This study highlights the need to improve treatment adherence. In a context of universal access to ART, combined with unavailability of VL and HIV-1 resistance testing particularly in remote areas, it is crucial that national programme managers make advocacy for better psychosocial support allowing treatment adherence with focus on capacity building and staffing of health personnel, especially at a decentralized level.

Phylogenetic analysis shows the predominance of CRF02_AG for each of the three participating countries. And, HIV-1 genotypes found in our study are concordant with those previously observed in Senegal [13], in Mali [19,25] and in Guinea-Conakry [34].

Our study has some limitations. First, to assess treatment adherence, a questionnaire was not provided to patients during the enrolment process. Second, the CD4 cell count at ART initiation was not available and sometime data relating to the treatment history (combination and substitution) too. Other limitations were the small number of patients recruited in some sites, the heterogeneous treatment duration of patients (ranging from 6 months to 11 years) and the recruitment process. For example, in Senegal, all samples received for routine virological monitoring and who met the inclusion criteria were considered. It was not the case in Mali and Guinea-Conakry, where the selection of recruitment sites was made based on the active file of patients. So, the results are not representative nationwide.

## Conclusions

Our data show a relatively high rate of patients in VF while among them an important proportion harboured wild-type viruses. This highlights a real need to reinforce treatment adherence and expand VL and resistance testing in remote areas. Regarding virological monitoring of patients followed in remote areas, these results provide additional evidence that the use of DBS samples could facilitate access to viral load and resistance testing for better care of patients in a context of limited resources.

## In memoriam

Tragically, our colleague and friend Dr Nestor Bangoura died before the publication of this article. We wish to honour his memory.
